# Dynamic RACH Partition for Massive Access of Differentiated M2M Services

**DOI:** 10.3390/s16040455

**Published:** 2016-03-30

**Authors:** Qinghe Du, Wanyu Li, Lingjia Liu, Pinyi Ren, Yichen Wang, Li Sun

**Affiliations:** 1 Department of Information and Communications Engineering, School of Electronic and Information Engineering, Xi’an Jiaotong University, Xi’an 710049, China; duqinghe@mail.xjtu.edu.cn (Q.D.); liwanyu@stu.xjtu.edu.cn (W.L.); wangyichen0819@mail.xjtu.edu.cn (Y.W.); lisun@mail.xjtu.edu.cn (L.S.); 2 Shaanxi Smart Networks and Ubiquitous Access Research Center, Xi’an 710049, China; 3 Department of Electrical Engineering and Computer Science, the University of Kansas, Lawrence, KS 66045, USA; lingjialiu@ittc.ku.edu

**Keywords:** M2M communications, overload control, preamble partition, access success probability, average access delay

## Abstract

In machine-to-machine (M2M) networks, a key challenge is to overcome the overload problem caused by random access requests from massive machine-type communication (MTC) devices. When differentiated services coexist, such as delay-sensitive and delay-tolerant services, the problem becomes more complicated and challenging. This is because delay-sensitive services often use more aggressive policies, and thus, delay-tolerant services get much fewer chances to access the network. To conquer the problem, we propose an efficient mechanism for massive access control over differentiated M2M services, including delay-sensitive and delay-tolerant services. Specifically, based on the traffic loads of the two types of services, the proposed scheme dynamically partitions and allocates the random access channel (RACH) resource to each type of services. The RACH partition strategy is thoroughly optimized to increase the access performances of M2M networks. Analyses and simulation demonstrate the effectiveness of our design. The proposed scheme can outperform the baseline access class barring (ACB) scheme, which ignores service types in access control, in terms of access success probability and the average access delay.

## 1. Introduction

With the rapid development of cellular networks, the M2M communications, known as an indispensable part of Internet of Things (IoT) [1,2], have received more and more research attention. Generally, M2M communications provide a flexible solution for many scenarios in IoT. On the one hand, M2M in LTE, relying on cellular networks as the infrastructure, provides the long-distance access option for IoT, such that better coverage can be achieved. On the other hand, M2M communications may also offer the possibility for short-range applications.

In general, a M2M communication system includes a large number of machine-type communication (MTC) devices that can communicate without human intervention [3,4,5,6,7,8,9]. However, when the number of MTC devices trying to transmit data to the eNB (eNodeB) is considerably large within a very short period of time, the radio access network (RAN) overload issue will arise accordingly. In such a condition, the network congestion [4,5] inevitably increases delays, causes packet loss and even leads to service interruption. To alleviate network congestion caused by RAN overload, several schemes [6] were proposed and studied, among which the access class barring (ACB) [7] scheme is currently regarded as a simple and popular solution in M2M networks. The key idea of the ACB scheme can be summarized as follows. Under the ACB scheme, the eNB broadcasts two parameters to all MTC devices. Based on the two parameters, each MTC device performs random access barring for itself. Particularly, each MTC generates a random number and compares the number with the threshold broadcast by the eNB. If the number is smaller than the threshold, it proceeds with the access attempt to the network; otherwise, it will back off for a random time period before attempting to access again. The ACB scheme is simple to implement, and thus, it receives wide attention.

Generally speaking, the ACB scheme has been shown to be beneficial when MTC devices can tolerate long access delays due to frequent collisions during random access. However, when there also exist delay-sensitive services, the ACB scheme might not work efficiently. In the literature, the extended access barring (EAB) scheme [8] takes the delay-sensitive devices into account. The basic idea of EAB can be summarized as: as long as EAB is activated in the case of network congestion, the delay-sensitive devices are enabled to send access requests while the delay-tolerant ones are disabled from doing so. This approach actually sacrifices the performance for delay-tolerant services. However, it is worth noting that in realistic M2M networks, the number of delay-sensitive services is far less than that of delay-tolerant ones. Clearly, benefiting delay-sensitive services too much would severely degrade the performances of delay-tolerant services and use the resource in a highly-inefficient way.

To address the aforementioned problems, this paper proposes an efficient scheme that performs dynamic allocation of the random access channel (RACH) resources to clustered MTC devices with differentiated delay requirements. Specifically, the proposed scheme can adjust the preamble partition ratio between the two given clusters. The original ACB scheme is further applied to each cluster for access attempts. The novelty and contribution of our work lie in the optimized partition solution, as well as the theoretical analyses. Simulation results show that compared to the baseline ACB scheme, the proposed scheme obtains significant improvement in access success probability and also achieves performance improvement in reducing access delay.

The remainder of this paper is organized as follows. Section 2 reviews the related work on existing research efforts and candidate solutions on M2M congestion control. Section 3 introduces the system framework and M2M traffic model. Section 4 proposes the dynamic RACH partition scheme in detail. Section 5 analyzes the performance for our proposed scheme. Then, Section 6 presents the simulation evaluations and compares our scheme with the baseline ACB scheme. Finally, the paper concludes with Section 7.

## 2. Related Work

Notably, the M2M communication system is a large-scale network with diverse applications and a massive number of interconnected machines. There are mainly two standards bodies pushing the standardization process of M2M communications: the Third Generation Partnership Project (3GPP) and the European Telecommunications Standards Institute (ETSI), which specified their respective M2M communication architectures. ETSI defined the service-oriented M2M network architecture that comprises the device-and-gateway domain and the network domain, but without the underpinning of particular wireless technologies [10]. Furthermore, the ETSI-M2M does not indicate the specifications for M2M area networks and details for the access and core networks. The 3GPP-M2M focuses on enhancing the cellular wireless networks. Consequently, the typical smartM2M and oneM2M architectures specified by ETSI are inclined to provide M2M services independent of the underlying networks. That is to say, the access overload issue is less serious in the ETSI-M2M architecture, although the management will be hard. In contrast, 3GPP categorizes MTC devices as a special type of cellular users with a low rate and priority, and the MTC devices need to connect to the base station for data transmission. Therefore, the centralized control is ready. However, when the population of MTC devices gets large, the congestion problem could be extremely severe. Since the applications of M2M networks typically require security and privacy, the centralized architecture still attracts the major attention. Consequently, in this paper, our efforts will be dedicated to M2M under the 3GPP architecture.

It is worth mentioning that in the early stage of proposals for MTC, direct connections between MTC devices, as well as multi-hop transmissions across MTC devices were also suggested [11]. However, these types of connections also often come with quality-of-service (QoS) requirements, such as delay requirements [12,13]. Then, these functions have been gradually carried out by the device-to-device (D2D) communications with cooperation and cognition capability [12,13,14,15], where adjacent devices can connect directly by reusing the cellular users’ spectrum in either an underlay [12,16], an overlay [17] or a hybrid style [13]. Multi-hop communications over D2D networks has also been attracting research attention [16,18].

Recently, comprehensive studies have been launched over the last few years to explore the RAN overload issue for M2M communications [19]. As suggested in [20], a huge volume of signaling and data flow will be yielded, easily causing severe congestions in random access network. Similar to wireless sensor networks (WSN) [21], massive M2M devices with burst data within a short period of time may also produce massive accesses [22], which result in radio access networks’ (RAN) overload issue. As M2M continues to burgeon rapidly, it is worth researching RAN overload control towards future ubiquitous IoT.

There are several research outcomes of solving the congestion problem [23]. Among all available solutions, ACB is recognized as an effective yet simple mechanism to regulate access in LTE/LTE-A networks [24,25]. Sixteen classes are defined, and several of them are reserved for high-priority cases. However, the access schemes for coexisting services with different priorities have not been specified. Towards this issue, several schemes have been proposed to adapt system parameters to the varying M2M network statuses. The work in [6] discussed several methods for modified ACB approaches: extended access barring (EAB), dynamic access barring (DAB) and cooperative ACB. The EAB scheme deals with differentiated services, which are divided into clusters with respective ACB parameters. However, different clusters still share the same access resources. The DAB scheme focuses on the dynamic adjustment of controlling parameters for ACB, while not addressing the differentiated services. We will show later that the partition of access preamble resources for differentiated services is the key to optimize the access performances, which have not been thoroughly studied in the literature.

Amokrane *et al.* [26] proposed a mechanism for congestion control in M2M networks. In this paper, congestion concerns both the radio access network and the mobile core network. The core idea of this work is to mitigate the MME/S-GWoverload by rejecting the MTC traffic at the radio access network [26]. This work can reduce the amount of signaling for MTC devices and can satisfy the desired resource utilization ratio in the core network. Lien *et al.* [11] comprehensively discussed ubiquitous massive access via 3GPP M2M communications. This work proposed an effective solution to provide QoS guarantees to facilitate M2M applications with hard timing constraints. Lo *et al.* [27] proposed a self-optimizing overload control (SOOC) scheme that can configure RA resources according to the load condition. A typical feature of the SOOC scheme is that it can collect useful information via overload monitoring. Then, it can dynamically adjust the RA resources. However, this work did not present simulation or experiment results for performance evaluations. Aside from the above work, congestion and overload control in M2M communications was also actively discussed in [28]. Some applications based on M2M communications were described in [29,30].

The work in [31] proposed a prioritized random access for alleviating RAN overload, which takes advantage of the joint design combing the dynamic access barring and virtual resource allocation. Yang *et al.* [32] proposed a backoff adjustment scheme, which can improve the performances for low congestion levels. A code-expanded RA mechanism was developed by [33], where RA slots can be assembled in groups. This approach can increase the amount of contention resource at the cost of extra energy.

As elaborated above, extensive research has been dedicated to the RAN-level contention mechanism. In summary, the essence of RAN overload control mechanisms includes: dispersing the load of random access to different time slots, barring the random access behaviors and tuning system parameters based on MTC access traffic [34]. Existing research outcomes on the ACB mechanism indicate that the ACB scheme is effective mainly for delay-tolerant devices. While for the EAB mechanism, although it takes delay-sensitivity into consideration and divides all devices into two groups, it cannot make full use of the preambles well. This is because it ignores the fact that the number of delay-sensitive services is far less than delay-tolerant ones in M2M networks. Moreover, it does not perform dynamic adjustment of preamble allocation to the two groups, and thus, it is hard to further enhance the performance. Note that in this paper, we use the term “group” and “cluster” for MTC devices exchangeably for presentation convenience.

## 3. System Model

This section firstly describes a typical M2M traffic model defined in 3GPP. Then, it briefly reviews the random access (RA) procedures in LTE-A. At last, the categories for M2M devices are specifically addressed.

### 3.1. System Architecture

**Figure 1:**
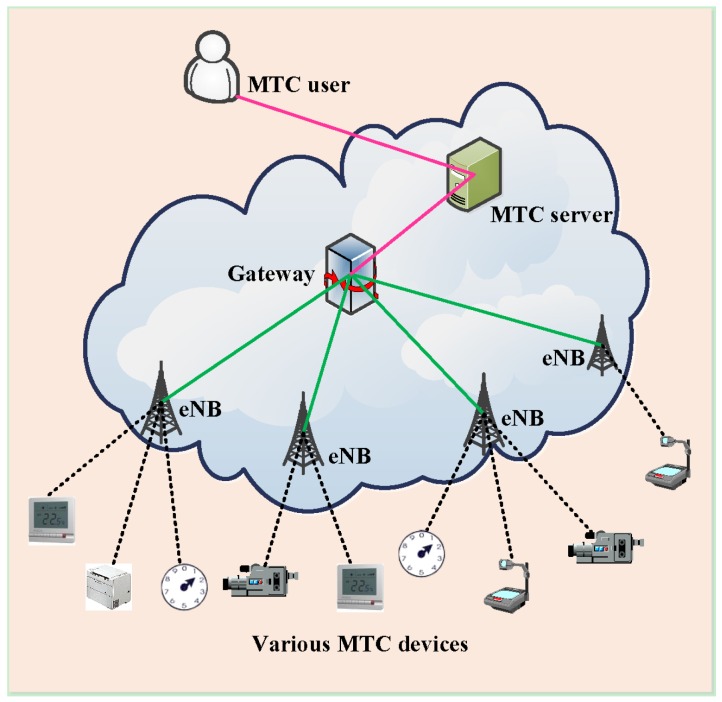
M2M communications in LTE-Advanced cellular networks towards IoT.

The network architecture of M2M communications in 3GPP is depicted in Figure 1, in which an MTC user can control various MTC devices via the MTC server. In the M2M network, various MTC devices access the eNB over wireless links. The eNB is responsible for collecting the data from MTC devices and forwards these data to the backhaul network and/or the Internet via a gateway for diverse applications. Notably, the M2M gateway ensures that M2M devices can interconnect with access networks. Clearly, the massive accesses of MTC devices to the eNB over wireless links form the major bottleneck and/or congestion in M2M networks, which, therefore, are the main focus of the work conducted in this paper. The work mainly concentrates on the multiple accesses by a large amount of MTC devices to the eNB. In order to help characterize the system architecture of the proposed scheme, here the motivation scenario in the case of the co-existence of delay-sensitive and delay-tolerant devices is specifically illustrated. In the scenario, delay-sensitive and delay-tolerant devices respectively correspond to one MTC application. The urban London scenario [31] is considered, and the delay-tolerant devices are from the application of smart meters. The delay-sensitive devices from the hospital e-care application are from the hospice beds from one of the largest hospitals in London. Obviously, the number of smart meters is quite huge, while the hospital e-care is very rare. When two types of services simultaneously attempt to access the network, the RAN overload issue appears. Meanwhile, the design of the resource allocation to realize the access control becomes very important, which is the work conducted in this paper.

### 3.2. Random Access Procedures

For MTC devices, we consider the typical random access procedures defined in LTE protocols [35], where all MTC devices compete for the available wireless resources. It is worth noting that in M2M networks, the contention-based random access procedure usually applies for delay-tolerant access requests. However, contention-free opportunistic access is also supported by pre-allocating particular resources, which are for delay-constrained requests. Because the amount of devices sending request with strict delay demands is quite small compared to delay-tolerant ones and the total number of such MTC devices is often huge, here we focus on the typical contention-based random access procedure, which consists of four steps [35] between MTC devices and the eNB. Moreover, the resource pre-allocation approach is very inflexible, and thus, not able to provide the requests with fine-grained delay requirements. Next, we elaborate on the detailed random access procedures as follows.

**Figure 2:**
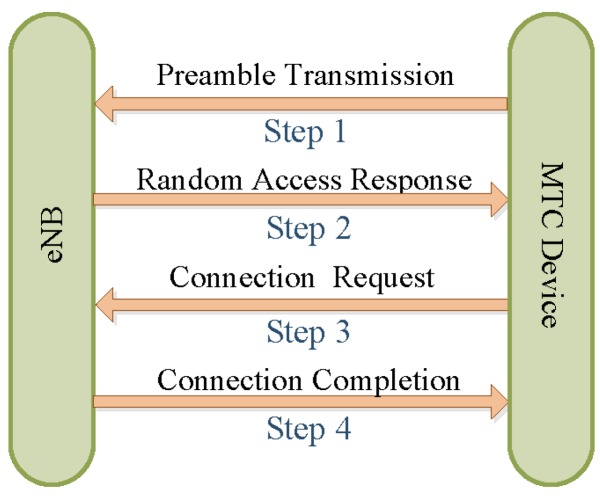
Random access procedures between the M2M device and eNodeB (eNB) in LTE.

When an MTC device attempts to access the network, it needs to send out an access request over the random access channel (RACH), which is comprised of several random access slots, which are used for the transmission of access requests. The length of the RA slot depends on the value of the configuration index. Relying on the existing protocols, the configuration index is valued as six, which means that in the RACH, there is an access opportunity every five milliseconds. In other words, the RACH finishes configuration every five milliseconds. Furthermore, there are in total 64 orthogonal available preambles. Only 54 of them are available for contention-based access, while the remaining 10 preambles are reserved for contention-free access. An access request is completed only if the four steps [31] are successfully finished, as shown in Figure 2 and Algorithm 1. However, note that the massive access attempts by sending the preambles occur in Step 1, which cause the majority of collisions. Thus, in this paper, we mainly concentrate on Step 1 to design the access control schemes.
**Algorithm 1**: Four steps of random access.1:**Step 1:** *Preamble transmission*Once an MTC device launches an access request to the RACH, it firstly selects a preamble of the RACH to transmit an access request. Under this condition, if two or more devices select the same preamble during the same slot, such that eNB is unable to decode any of the preambles, a collision occurs.2:**Step 2:** *Random access response (RAR)*For each successfully-decoded preamble, the eNB computes an identifier and then transmits an RAR to the UEdevices.3:**Step 3:** *Connection request*The M2M device transmits a connection request message with a UE identifier to the eNB.4:**Step 4:** *Contention completion*Upon reception of a connection request in Step 3, the eNB transmits a contention resolution message as an answer to Step 3. Therefore, a device that does not receive Step 4 indicates a failure in the contention completion and requests a new access attempt.


Before proceeding further, we need to explain the fundamental mechanism of the ACB mechanism and to define the corresponding parameters used in this paper. The ACB scheme [11] was proposed for random access control of MTC devices. Particularly, the eNB broadcasts two parameters: an access barring factor, denoted by ac_barringfactor, and an access barring time, denoted by ac_barringtime. Each MTC device attempting to access the network generates a uniformly-distributed random variable *q*, 0 ⩽ *q* ⩽ 1. If *q* ⩽ ac_barringfactor, the MTC device continues with the random access procedures. Otherwise, it is barred for a random time duration based on ac_barringtime by using Equations (24) and (25) to retry the access.

### 3.3. Categories of M2M Devices

According to the comprehensive research [36], in the real scene where delay-sensitive and delay-tolerant devices coexist, although the occurrence of delay-sensitive devices, such as the hospital e-care, is very rare, the delay-sensitive devices are strict with delay, and they need instant processing. For the application in hospital e-care, the maximum tolerant delay is five milliseconds, since in a real system, the data become useless after that. On the other hand, delay-tolerant devices, such as smart grids [11], can tolerate several seconds or even minutes, and a great majority of devices is subject to this category.

## 4. Resource Partition Scheme for M2M Networks

The proposed scheme solves the RAN overload problem in the scenarios of the co-existence of delay-sensitive and delay-tolerant devices. In general, the proposed scheme comprehensively takes the resource allocation and the access control into account for the scenarios where two types of services co-exist in M2M networks. Specifically, the proposed architecture is composed of several main moves: firstly, in order to effectively provide quality of service (QoS) for two types of MTC devices mentioned above, we consider that MTC devices are classified into two clusters on the basis of delay requirements. Secondly, the vital conceptual design is achieved by dynamically adjusting the preamble partition between two given clusters. Thirdly, devices from two clusters adopt the ACB mechanism to access the network, respectively.

**Figure 3:**
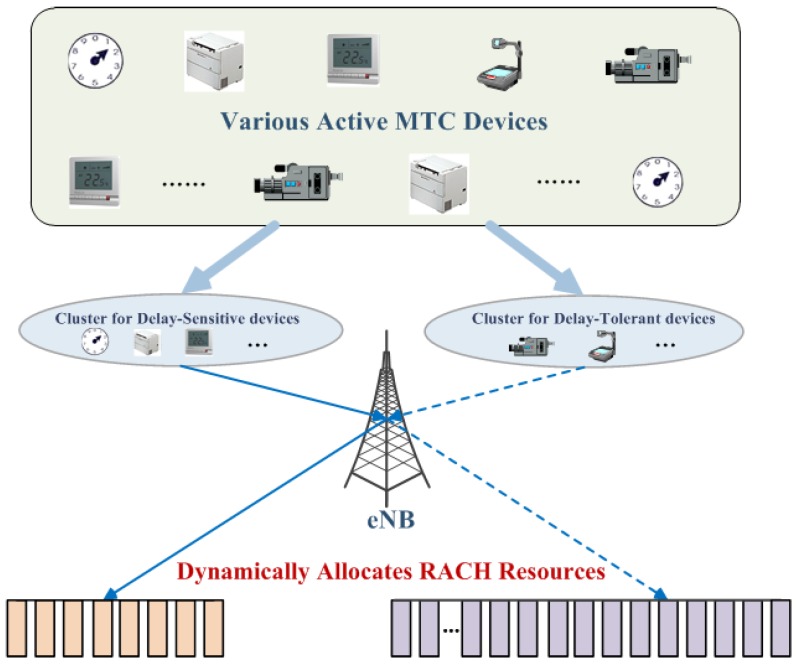
The diagram of the conceptual design.

According to the proposed principle discussed above, as depicted in Figure 3, this paper presents the implementations at length afterwards.

### 4.1. Clustered Structure

Due to the lower incidence of delay-sensitive services [37], the delay-sensitive devices utilize the preamble resources occasionally, which results in smaller traffic loads comparing to the delay-tolerant ones. Considering this, we divide those devices that are attempting to access the network into two clusters according to their requirements of delay. In the actual implementation of our scheme, we pre-categorize the attempting devices into two clusters. In other words, once the total number of devices is given, the devices would be automatically divided into two clusters.

### 4.2. Dynamic Adjustment of Preamble Partition

After dividing the attempting devices into two clusters, the eNB dynamically determines the RA preamble partition between two clusters before accessing the network. We propose a feasible scheme to obtain the preamble partition. To formulate such a random access problem, we pre-define the variables as follows.

In a certain RA slot, we denote *A_s_* as the number of delay-sensitive devices, *A_n_* as the number of delay-tolerant ones and *A_i_* as the total number of the active MTC devices. Namely, *A_s_* + *A_n_* = *A_i_*. Then, we define the preamble partition as:

(1) $$\beta =\frac{M_{s}}{M_{n}}$$

where *M_s_* and *M_n_* respectively represent the preambles allocated for delay-sensitive and delay-tolerant devices.

According to the configuration of RACH, we know:

(2) $$M_{s}+M_{n}=c$$

Connecting Equation (1) with Equation (2) where *c* = 54, we solve:

(3) $$M_{s}=\frac{c\beta }{1+\beta }$$

(4) $$M_{n}=\frac{c}{1+\beta }$$

Moreover, we denote *f_s_* and *f_n_* as the ac_barringfactors of two clusters, since we adopt the ACB scheme to access the network discussed in Section 4.3.

Since every MTC device selects the preamble randomly from the available resources pool, so collisions would occur if at least one MTC device transmits the same preamble. Moreover, according to [35], the contention-based RA procedure adopts a slotted-aloha as the access protocol, where the number of preambles available is analogous to the number of slots. In this way, the access success probability could be calculated by:

(5) $$P_{s}=e^{-\;\frac{N}{P}}$$

where *N* is the total number of devices and *P* is the number of preambles available within an RA slot. On the basis of the illustration above, the number of devices successfully completing the access attempts *S_N_* is defined as:

(6) $$S_{N}=A_{s}f_{s}\times e^{-\;\frac{A_{s}f_{s}}{M_{s}}}+A_{n}f_{n}\times e^{-\;\frac{A_{n}f_{n}}{M_{n}}}$$

Then, substitute Equations (3) and (4) into Equation (6), and we can easily get:

(7) $$S_{N}=A_{s}f_{s}\times e^{-\;\frac{A_{s}f_{s}\left(1+\beta \right)}{c\beta }}+A_{n}f_{n}\times e^{-\;\frac{A_{n}f_{n}\left(1+\beta \right)}{c}}$$

Obviously, our ultimate purpose is to maximize *S_N_*. However, from the curve of *S_N_ versus β*, we can observe that when *β* is relatively smaller, *S_N_* probably reaches its maximum, which implies that *S_N_* could reach its maximum in the case of a suitable value of *β*. In other words, we can maximize the *S_N_* by limiting the number of preambles allocated for delay-sensitive devices, which relates to the number of sensitive devices successfully accessing the network. Consequently, by intentionally setting the bounds of the sensitive devices successfully accessing the network, our scheme is formulated as the optimization problems depicted in Equation (8).

(8) $$\left\{\begin{array}{ccc} \max_{\beta } & \left\{A_{s}f_{s}\cdot e^{-\frac{A_{s}f_{s}}{c}\left(1+\frac{1}{\beta }\right)}+A_{n}f_{n}\cdot e^{-\frac{A_{n}f_{n}\left(1+\beta \right)}{c}}\right\} & \text{if}\;A_{s}f_{s}\in \left(0,1\right]\\ \max_{A_{s}f_{s}e^{-\;\frac{A_{s}f_{s}}{c}\left(1+\frac{1}{\beta }\right)}⩾1} & \left\{A_{s}f_{s}\cdot e^{-\frac{A_{s}f_{s}}{c}\left(1+\frac{1}{\beta }\right)}+A_{n}f_{n}\cdot e^{-\frac{A_{n}f_{n}\left(1+\beta \right)}{c}}\right\} & \text{if}\;A_{s}f_{s}\in \left(1,3\right)\\ \max_{A_{s}f_{s}e^{-\frac{A_{s}f_{s}}{c}\left(1+\frac{1}{\beta }\right)}\geq 2} & \left\{A_{s}f_{s}\cdot e^{-\frac{A_{s}f_{s}}{c}\left(1+\frac{1}{\beta }\right)}+A_{n}f_{n}\cdot e^{-\frac{A_{n}f_{n}\left(1+\beta \right)}{c}}\right\} & \text{if}\;A_{s}f_{s}\in \left[3,+\infty \right) \end{array}\right.$$

The objective function in Equation (8) maximizes the total number of devices successfully completing the access procedure under three restrictions of *β*. Then, we analyze the formulated optimization problems and correspondingly find their optimal solutions (denoted as β*), respectively.

**Case 1:** *A_s_f_s_* ∈ (0, 1]

When *A_s_f_s_* ∈ (0, 1], our formulated problem becomes an unconstrained optimization problem. First, we get the first-order derivative of the objective function depicted in Equation (9):

(9) $$\frac{A_{s}^{2}f_{s}^{2}}{c\beta^{2}}\times e^{-\;\frac{A_{s}f_{s}\left(1+\beta \right)}{c\beta }}-\frac{A_{n}^{2}f_{n}^{2}}{c}\times e^{-\;\frac{A_{n}f_{n}\left(1+\beta \right)}{c}}\equiv 0$$

and let Equation (9) be equal to zero. By simplifying Equation (9), we have:

(10) $$\frac{A_{s}f_{s}}{c}\left(1+\frac{1}{\beta }\right)-\frac{A_{n}f_{n}}{c}\left(1+\beta \right)+2\log\beta =\log\left(\frac{A_{s}^{2}f_{s}^{2}}{A_{n}^{2}f_{n}^{2}}\right)$$

Since there is no closed solution for Equation (10) through using Mathematica, we decide to find the approximate solution by means of numerical analysis. We adopt the **Newton iteration algorithm** depicted in Algorithm 2 to find the approximate solution β*.
**Algorithm 2**: Newton iterative algorithm.1:**Initialization:** endow *A_s_*, *A_n_*, *f_s_*, *f_n_* with any feasible values and then set the initial value of iteration *β*_0_;2:**Iteration:** taking *β*_0_ as the iteration initial value and 10^−5^ as the given precision, the Newton iterative algorithm is used to search for the first approximate solution *β*_1_;3:**Recursion:** take *β*_1_ as the iteration initial value and 10^−5^ as the given precision to get the second approximate solution *β*_2_, and repeat the steps above to get the solution set {*β*_1_, *β*_2_, … *β_m_*};4:**Confirmation:** substitute {*β*_1_, *β*_2_, … *β_m_*} into the objective function in turn and select the β* for maximizing the objective function;5:**End**


**Case 2:** *A_s_f_s_* ∈ (1, 3)

Different from **Case 1**, the problem under this case becomes an optimization problem with an inequality constraint. First, we determine the feasible domain described as:

(11) $$D_{1}=\left\{\beta \left|\beta ⩾\frac{A_{s}f_{s}}{c*\log\left(A_{s}f_{s}\right)-A_{s}f_{s}}\right.\right\}$$

and then determine the monotonicity of the objective function in the feasible domain. Since the objective function is monotonically decreasing within the feasible domain, the maximum value of the objective function is gained at the boundary of the feasible region, namely:

(12) $$\beta^{*}=\frac{A_{s}f_{s}}{c*\log\left(A_{s}f_{s}\right)-A_{s}f_{s}}$$

**Case 3:** *A_s_f_s_* ∈ [3, +∞)

Similar to **Case 2**, the feasible domain under this case is illustrated as:

(13) D2=ββ⩾Asfsc∗log(AsfsAsfs22)−Asfs

Similar to the analysis in **Case 2**, the optimal β* is expressed as:

(14) β∗=Asfsc∗log(AsfsAsfs22)−Asfs

In summary, according to the conceptual design discussed above in a certain slot, once we have known the *A_s_*, *A_n_*, *f_s_*, *f_n_*, the eNB would obtain the optimal preamble partition based on the proposed scheme.

### 4.3. Access Class Barring for Two Clusters Respectively

After obtaining the preamble partition, the devices belonging to different clusters adopt the ACB scheme respectively to access the network. According to the ACB mechanism, we propose that the ac_barringtime of two clusters is configured as the same. In order to simplify our proposed architecture, the connection between two ac_barringfactors would be devised as the following linear correlation: *f_n_* = *p* + *q* ∗ *f_s_* where *f_s_* ∈ (0, 1) and the value of *p* and *q* should satisfy *f_n_* ∈ (0, 1). In Section 6, we will show the parameter settings and illustrate the proofs in detail.

## 5. Analysis of the Resource Partition Scheme

In this part, we mainly address the theoretical performance analysis of the proposed scheme. Before that, the estimation of the access loads should be taken into consideration, since we assume that the traffic loads during one slot are pre-known in the proposed scheme. As previously mentioned, once obtaining β* in a slot, the eNB would allocate the corresponding number of preambles to the two clusters based on the proposed preamble partition scheme. Based on the number of allocated preambles and the collision status in each slot, we can apply a Markov-based approach, which was developed in [38], to well estimate the traffic load of each cluster dynamically.

Next, before analyzing the performance of the proposed scheme, we consider the following performance indexes:

(**a**) *Access success probability (ASP)*, defined as the ratio between total devices completing the RA procedures and the total access attempts within the same slot.

(**b**) *Preamble collision probability (PCP)*, defined as the ratio between the number of preamble collision and the total preambles transmitted within the same slot.

(**c**) *Average access delay (AAD)*, defined as the average delay between the first attempt and the completion of all RA procedures for the devices that successfully access the network.

### 5.1. Analysis of Access Success Probability

Under the assumptions and notations defined in Section 4, we will subsequently analyze the performance from three aspects. Additionally, so as to compare with the simulation results in Section 6, we suppose that the ratio of the number for two clusters is denoted as *γ*. Then, we can get *A_n_*/*A_s_* = *γ*, as well as *A_s_* + *A_n_* = *A_i_*. Accordingly, *A_s_* and *A_n_* are expressed as follows:

(15) $$A_{n}=\frac{\gamma A_{i}}{1+\gamma }$$

(16) $$A_{s}=\frac{A_{i}}{1+\gamma }$$

Next, once we have solved the β* in the proposed scheme, we would obtain the number of devices successfully completing the access attempts *S_N_* in a certain slot by substituting β* into Equation (7), described as:

(17) $$S_{N}=A_{s}f_{s}\times e^{-\;\frac{A_{s}f_{s}\left(1+\beta^{*}\right)}{c\beta^{*}}}+A_{n}f_{n}\times e^{-\;\frac{A_{n}f_{n}\left(1+\beta^{*}\right)}{c}}$$

Thus, the ASP in a certain slot is calculated by:

(18) $$P_{S}=\frac{A_{s}f_{s}\times e^{-\;\frac{A_{s}f_{s}\left(1+\beta^{*}\right)}{c\beta^{*}}}+A_{n}f_{n}\times e^{-\;\frac{A_{n}f_{n}\left(1+\beta^{*}\right)}{c}}}{A_{i}}$$

Then, substituting Equations (15) and (16) into Equation (18), the expression Equation (18) can be simplified as:

(19) $$P_{S}=\frac{f_{s}}{1+\gamma }\times e^{\frac{-f_{s}A_{i}}{(1+\gamma )c}\left(1+\frac{1}{\beta^{*}}\right)}+\frac{\gamma f_{n}}{1+\gamma }\times e^{\frac{-\gamma A_{i}f_{n}}{(1+\gamma )c}\left(1+\beta^{*}\right)}$$

In accordance with the simulation setup in Section 6, here we generally present the parameter settings in advance. First we devise the connection between two ac_barringfactors as: $f_{n}=0.6+\frac{1}{3}*f_{s}$, which are explained in Section 6.1. Moreover, the ratio of the number for two clusters is defined as *γ* = 9. The traffic model is devised as a *β*-distribution within 10 s. In this way, from a realization example of the number of MTC arrivals within 10 s, we can see that the number of access attempts falls within the range from one to 50. It is worth noting that in our analysis, we set the lower bound for *A_i_* as one instead of zero, which is the actual minimum just to avoid the denominator in Equation (18) being zero, while not affecting the analysis results. On the other hand, we set the maximum value for *A_i_* as 50.

**Figure 4:**
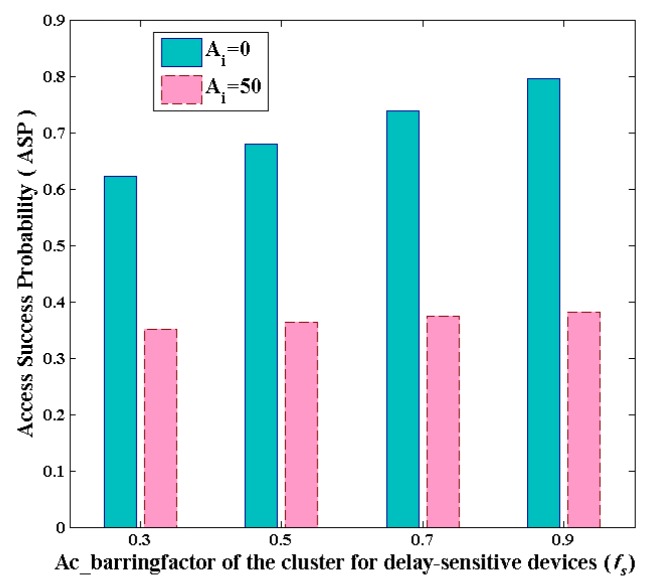
The access success probability (ASP) of two extreme cases *versus f_s_* during one slot.

Since the number of access attempts during every slot is rand, here we typically choose two extremes to present the analysis on ASP. One is the maximum number of access attempts; the other is the minimum one. Here, we depict the relationships between *f_s_* and *P_S_* in two extreme cases respectively in Figure 4. It can be seen that the ASPs of two extreme scenarios are both rising with the increase of ac_barringfactor. Meanwhile, the larger the access load is, the worse the access condition becomes.

### 5.2. Analysis of Preamble Collision Probability

Similarly, on the basis of the derivation in ASP performance, we can analyze the preamble collision probability along this way. Inspired by Equations (5) and (7), we present the PCP during one slot defined as *P_C_*:

(20) $$P_{C}=\frac{A_{s}f_{s}\cdot \left(1-e^{\frac{-A_{s}f_{s}}{c}\left(1+\frac{1}{\beta^{*}}\right)}\right)+A_{n}f_{n}\cdot \left(1-e^{\frac{-A_{n}f_{n}}{c}\left(1+\beta^{*}\right)}\right)}{A_{s}f_{s}+A_{n}f_{n}}$$

After similar arrangements and simplifications, Equation (20) is described as Equation (21):

(21) $$P_{C}=1-\frac{\frac{\gamma f_{s}}{1+\gamma }\cdot e^{\frac{-\gamma f_{s}A_{i}}{(1+\gamma )c}\left(1+\frac{1}{\beta^{*}}\right)}+\frac{f_{n}}{1+\gamma }\cdot e^{\frac{-A_{i}f_{n}}{(1+\gamma )c}\left(1+\beta^{*}\right)}}{\frac{\gamma f_{s}}{1+\gamma }+\frac{f_{n}}{1+\gamma }}$$

**Figure 5:**
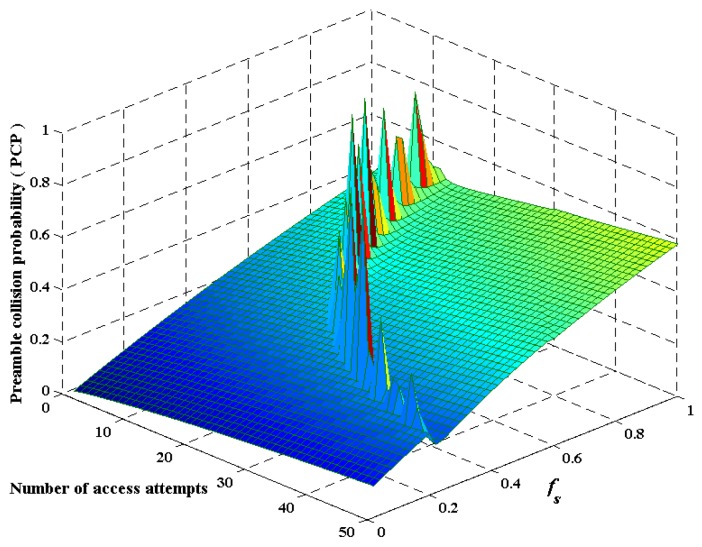
Collision probability varies with the synchronously change of *A_i_* and *f_s_* during one slot.

We depict the three-dimensional curve, which presents the variation tendency of *P_C_* when *A_i_* and *f_s_* synchronously change in Figure 5. During a certain slot, we can observe that the PCP is increasing as *f_s_* raises. Furthermore, the PCP also climbs with the rising of the total attempts during one slot. Here, it is worth noting that there are several *singular points* changing suddenly. According to the conceptual design discussed in Section 4, *A_s_f_s_* = 1 is taken as the “critical condition” distinguishing between two different ways of analysis, as well as solving. In Figure 5, it is verified that *singular points* approximately appeared near $A_{s}f_{s}=\frac{A_{s}f_{s}}{1+\gamma }=1$.

### 5.3. Analysis of Average Access Delay

The access delay includes two main parts comprised of the random backoff *Tbarred* in Equations (24) and (25) due to ac_barringfactor, as well as the fixed backoff due to collisions. Since there is a random number (defined as *rand*) ranging from zero to one in *Tbarred*, here we consider the potential upper bound and lower bound during one slot.

First, in the case of the lower limit, we assume *rand = 0* in Equations (24) and (25), and the AAD (seconds) is described as:

(22) $$\begin{array}{ccc} D_{lower}= & A_{s}\left(1-f_{s}\right)\times \left(0.7+0.6*0\right)*4+A_{n}\left(1-f_{n}\right)\times \left(0.7+\frac{0.6*0}{f_{n}}\right)*4 & +P_{C}\left(A_{s}f_{s}+A_{n}f_{n}\right)\times 5\times 10^{-3} \end{array}$$

where 5 × 10^−3^, denoting five milliseconds, represents the fixed backoff due to collisions. That is to say, once collision occurs, the access backs off for 5 ms.

Then, in the case of the upper bound, we assume *rand = 1*, and similarly, the AAD (seconds) is expressed as:

(23) $$\begin{array}{ccc} D_{upper}= & A_{s}\left(1-f_{s}\right)\times \left(0.7+0.6*1\right)*4+A_{n}\left(1-f_{n}\right)\times \left(0.7+\frac{0.6*1}{f_{n}}\right)*4 & +P_{C}\left(A_{s}f_{s}+A_{n}f_{n}\right)\times 5\times 10^{-3} \end{array}$$

## 6. Simulation Evaluation

In this section, we present the setup of the simulation parameters and scenarios, as well as the simulation results that demonstrate the advantageous performance of the proposed scheme for solving the RAN overload problems.

### 6.1. Simulation Setup

**Figure 6:**
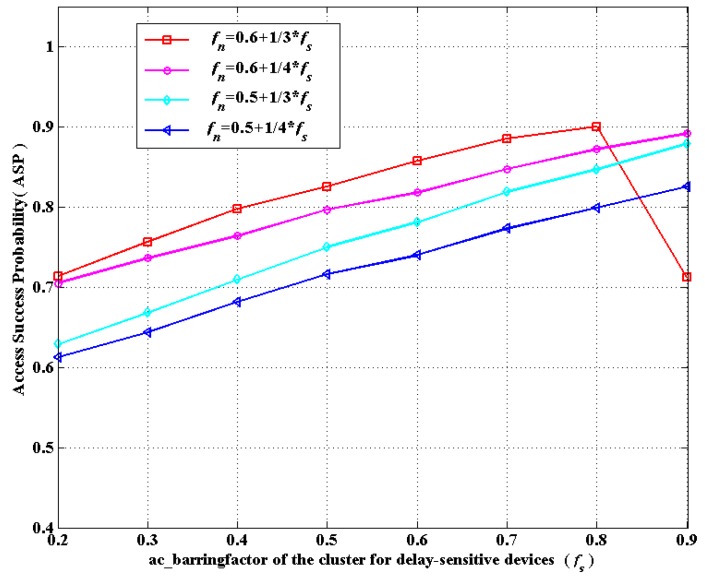
The ASP *versus* the linear correlation of *f_s_* and *f_n_*.

**Figure 7:**
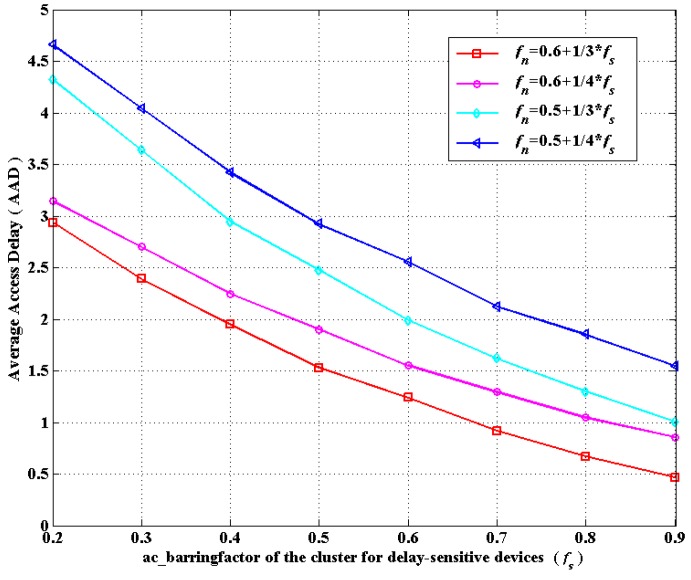
The average access delay (AAD) *versus* the linear correlation of *f_s_* and *f_n_*.

According to 3GPP protocols [39], the detailed LTE-A simulation parameters [40], as well as the typical traffic model [34] are depicted in Table 1. In order to evaluate our proposed scheme, we need to illustrate the basic parameters in the common ACB mechanism available in the proposed scheme.

(**a**)As discussed in Section 4, we first define the proportion between sensitive devices and non-sensitive devices as 1:9 due to the low incidences of sensitive devices according to our comprehensive research.

(**b**) As for the two parameters broadcast in the ACB mechanism, we shall assume that the ac_barringtime of two clusters is equal to 4 s. According to [41], in case of being barred, a sensitive device re-attempts access after a delay of:

(24) Tbarred=(0.7+0.6∗rand)∗ac_barringtime

where *rand* represents a random number uniformly drawn from [0, 1). Yet, the non-sensitive device re-attempts access after a delay of:

(25) $$Tbarred=(0.7+\frac{0.6*rand}{ac\_barringfactor})*ac\_barringtime$$

(**c**) According to the ACB mechanism, ac_barringfactor ranges from zero to one. Hence, we shall select suitable values for *p* and *q* in *f_n_* = *p* + *q* ∗ *f_s_* in order to satisfy *f_n_* ∈ (0, 1) when *f_s_* is given in advance. Accordingly, through simulations in Figure 6 and Figure 7, on the basis of the considerations of ASP and AAD, we choose the linear correlation of *f_s_* and *f_n_* as: $f_{n}=0.6+\frac{1}{3}*f_{s}$, which can achieve the optimal overall performance. Notably, there is a sharp decline in Figure 6 when *f_s_* changes from 0.8 to 0.9 in the case of $f_{n}=0.6+\frac{1}{3}*f_{s}$. This is because when *f_s_* changes from 0.8 to 0.9, *f_n_* changes from 0.87 to 0.9, which means nearly 90% of delay-tolerant devices would compete for access. In addition, due to the huge number of delay-tolerant devices, devices competing for access do not successfully access the network more easily compared to the situations for fewer devices. The more competing devices there are, the more frequent the collisions are, which results in lower ASP. Thus, there is a sharp decline as *f_s_* varies from 0.8 to 0.9. Therefore, setting *f_s_* as 0.9 is practically meaningless in real scenarios. Consequently, our simulation is conducted in $f_{n}=0.6+\frac{1}{3}*f_{s}$.

(**d**) The baseline scheme we use for performance comparison is the original ACB scheme, since it is the currently-suggested solution for RAN overload in M2M networks by 3GPP. Notably, the baseline ACB scheme neither divides the MTC devices into cluster groups nor partitions the preambles.

### 6.2. Simulation Results

**Figure 8:**
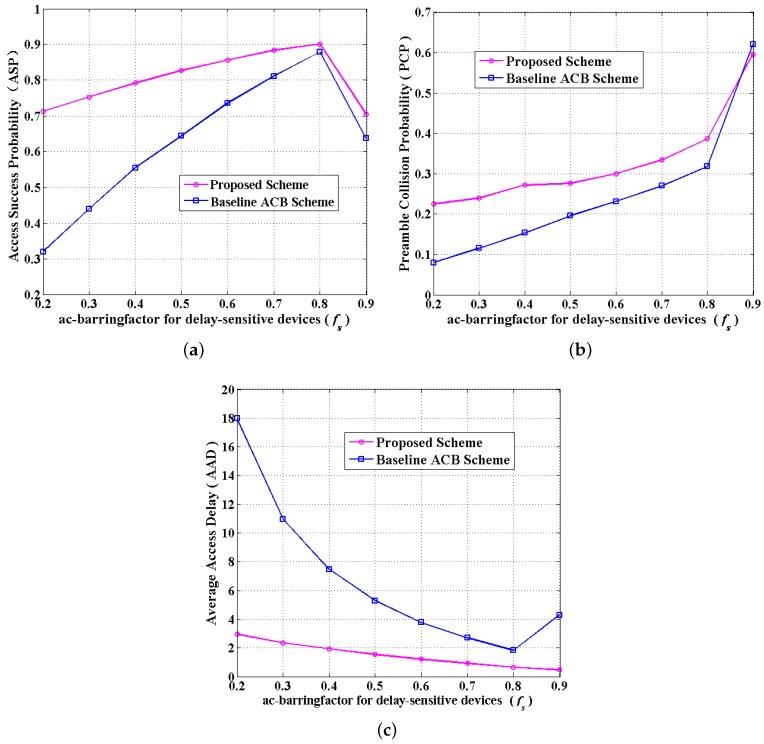
Performance comparison between the proposed scheme and the baseline access class barring (ACB) scheme. (**a**) The ASP *versus* the ac_barringfactor of the cluster for delay-sensitive devices; (**b**) the preamble collision probability (PCP) *versus* the ac_barringfactor of the cluster for delay-sensitive devices; (**c**) the AAD *versus* the ac_barringfactor of the cluster for delay-sensitive devices.

In Figure 8, we demonstrate the simulation results in terms of the overall ASP, PCP and AAD under the proposed scheme compared to the baseline ACB scheme. Figure 8a,c shows the performance of ASP and AAD *versus f_s_* under the proposed scheme and the baseline ACB scheme. Compared to the baseline ACB scheme, the proposed scheme yields much better performance in improving the overall access success probability, as well as reducing the average access delay. From the general trends in Figure 8a, two curves almost simultaneously climb up when *f_s_* varies from 0.2 to 0.8 and drop when *f_s_* varies from 0.8 to 0.9. Furthermore, we can observe from Figure 8a that there is a large gap between two curves when *f_s_* is smaller than 0.7, and the gap narrows when *f_s_* = 0.8. This is because when *f_s_* changes from 0.8 to 0.9, *f_n_* changes from 0.87 to 0.9, which means nearly 90% of delay-tolerant devices would compete for access. In addition, due to the huge number of delay-tolerant devices, devices competing for access do not successfully access the network more easily compared to the situations for fewer devices. The more competing devices there are, the more frequent the collisions are, which results in lower ASP in Figure 8a and longer access delay in Figure 8c. Similarly, two curves in Figure 8c drop when *f_s_* changes from 0.2 to 0.8, and the gap between them narrows continuously, as well a slight increase occurs in two curves when *f_s_* varies from 0.8 to 0.9. Obviously, the optimal value for *f_s_* can be adopted as 0.8 for the proposed scheme to obtain high ASP and lower AAD.

Instead, the preamble collision probability of the proposed scheme performs higher PCP for about 10%, while the growth falls when *f_s_* rises in Figure 8b, which is accounted for by the rise of the number of non-barred MTC devices when *f_s_* is larger. Consequently, in order to visually present the trade-off between access success probability and collision probability, here we introduce a weighting factor denoted as *ω* and a new indicator defined as the comprehensive parameter index (CPI). Namely, the CPI can be expressed as:

(26) $$CPI=\omega \cdot P_{S}+\left(1-\omega \right)\cdot P_{C}$$

**Figure 9:**
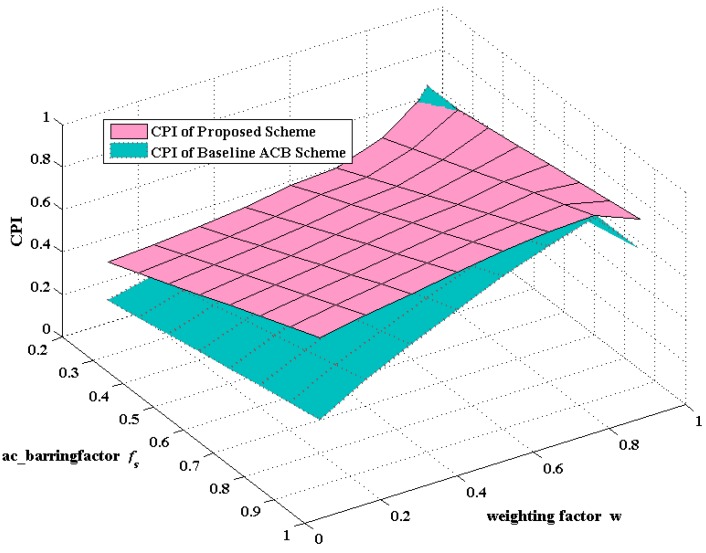
The comprehensive parameter index (CPI) varies with the synchronous change of *ω* and *f_s_* between the proposed scheme and the baseline ACB scheme.

Figure 9 depicts the three-dimensional curve, which demonstrates the variation tendency of CPI with the synchronous change of *f_s_* and *ω*. Furthermore, Figure 9 reveals that the CPI of the proposed scheme is higher than that of the ACB scheme with the variation of weighting factor *ω* when fixing the given *f_s_*.

**Figure 10:**
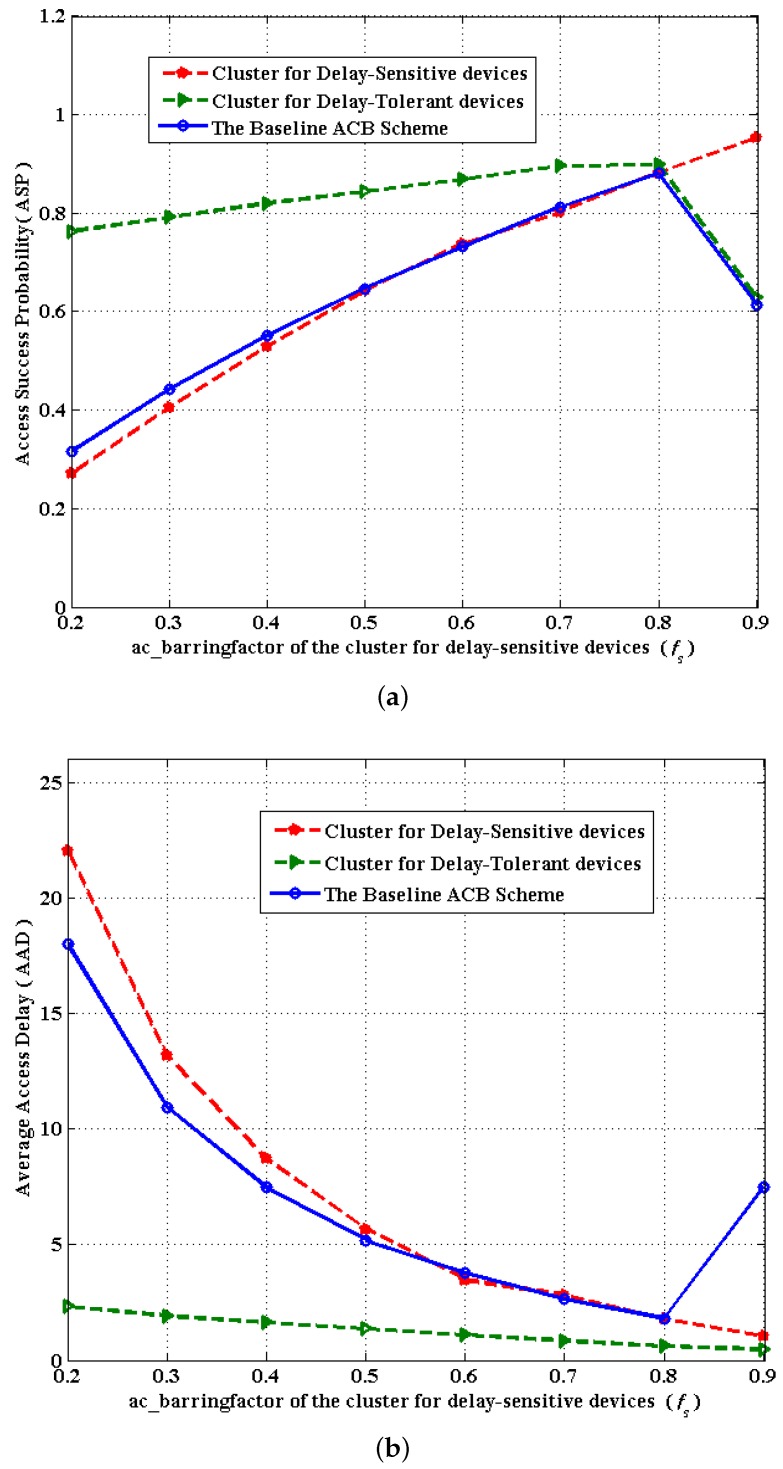
Performance comparison between two clusters for delay-sensitive and delay-tolerant devices and the baseline ACB scheme. (**a**) The ASP *versus f_s_* between two clusters and the baseline ACB scheme; (**b**) the AAD *versus f_s_* between two clusters and the baseline ACB scheme.

In comparison, Figure 10 also presents the performance of two clusters for delay-sensitive and delay-tolerant devices separately, which shows that our proposed scheme achieves higher performance without sacrificing any kinds of services. From Figure 10, we can observe that when *f_s_* is larger than 0.6, the proposed scheme provides a slight effect on delay-sensitive services, while greatly improving the performance of delay-tolerant services in terms of ASP, which significantly increases the overall performance. In Figure 10a, the cluster for delay-sensitive devices yields a slightly lower ASP than when *f_s_* is smaller than 0.5, which makes no difference, since *f_s_* is set larger than 0.5 in a real system. Similarly, Figure 10b demonstrates that when *f_s_* is larger than 0.5, the proposed scheme provides a slight effect on delay-sensitive services, while effectively reducing the AAD for delay-tolerant services, which significantly increases the overall performance. Notably, Figure 10a,b comprehensively demonstrates that the cluster for delay-tolerant devices yields more effective performance than the baseline ACB scheme.

## 7. Conclusions

In this paper, we firstly presented the RAN overload issue caused by massive devices attempting to access the eNB in the M2M communication architecture. As for the scenario where delay-sensitive and delay-tolerant devices coexist, we then proposed a mechanism for access control to jointly guarantee the RAN overload requesting from differentiated services. Based on the loading condition for attempts to access from two kinds of devices, our proposed scheme dynamically allocates the preambles to respectively accommodate both sides. In our work, the core is to devise an optimal preamble partition for both sides in order to realize the maximum access success probability during each slot. We then theoretically analyzed the performance from three aspects and provided different forms of the analysis results. In addition, computer simulations are conducted to demonstrate that our proposed scheme has performance superiority over the baseline ACB scheme in terms of overall access success probability and access success delay.

## Figures and Tables

**Table 1:** Basic simulation parameters.

Parameter	Settings
Cell bandwidth	5 MHz
Number of M2M devices	30,000
Attempts’ distribution	Beta distribution
Distribution period	10 s
PRACH configuration index	6
Number of preambles for contention-based RA	54
Ra-ResponseWindowSize	5 sub-frames
Backoff indicator	5 ms
